# Patterns, Timing, and Survival of Recurrence After Surgery for Esophageal and Junctional Adenocarcinoma in the European Multicentre ENSURE Study

**DOI:** 10.1245/s10434-026-19153-8

**Published:** 2026-04-06

**Authors:** Lucas Goense, Jessie A. Elliott, Sheraz R. Markar, Fredrik Klevebro, Asif Johar, Styliani Mantziari, Pernilla Lagergren, Giovanni Zaninotto, Richard van Hillegersberg, Mark I. van Berge Henegouwen, Magnus Nilsson, George B. Hanna, John V. Reynolds, ENSURE Study Group

**Affiliations:** 1 Department of Surgery, University Medical Center Utrecht, Utrecht University, Utrecht, The Netherlands; 2 Trinity St. James’s Cancer Institute, Trinity College Dublin, St. James’s Hospital, Dublin, Ireland; 3 Nuffield Department of Surgical Sciences, University of Oxford, Oxford, UK; 4 Department of Clinical Science Intervention and Technology (CLINTEC), Department of Upper Abdominal Diseases, Karolinska Institutet, Karolinska University Hospital, Stockholm, Sweden; 5 Department of Molecular Medicine and Surgery, Karolinska Institutet, Stockholm, Sweden; 6 Lausanne University Hospital CHUV, Lausanne, Switzerland; 7 Department of Surgery and Cancer, Imperial College London, St. Mary’s Hospital, London, UK; 8 Department of Surgery, Amsterdam UMC, University of Amsterdam, Amsterdam, The Netherlands; 9 Cancer Center Amsterdam, University of Amsterdam, Amsterdam, The Netherlands

**Keywords:** Esophageal adenocarcinoma, Recurrence patterns, Post-recurrence survival, Recurrence-free interval, Metastatic sites, Locoregional recurrence

## Abstract

**Background:**

Following surgery for esophageal cancer, approximately half of patients develop disease recurrence. Analyzing recurrence site, timing, and their impact on postrecurrence survival can guide refinements in postoperative surveillance and therapy. This study aimed to describe the pattern and timing of esophageal cancer recurrence and assess survival patterns by recurrence location.

**Methods:**

This study included patients from the ENSURE study who underwent resection for esophageal and junctional adenocarcinoma between 2009 and 2015 across 20 centers in Europe and Canada. Only patients with disease recurrence were included in the final analysis. Sites of first recurrence were stratified into different groups and survival outcomes postrecurrence detection were estimated using Kaplan-Meier curves.

**Results:**

Of 3,299 patients who underwent surgery for esophageal adenocarcinoma, 1,310 patients developed recurrence and had sufficient follow-up data available. First recurrence was detected at multiple distant sites (n = 469; 37.3%) or at a single distant site (n = 463; 33.7%), whereas isolated local recurrence occurred in 189 (14.4%) patients. Recurrence in the liver-only presented significant earlier (median 9.0 months), whereas recurrence in lung-only (15.2 months) and local-only recurrence (17.8 months) were observed later. Patients with multiple-sites and liver-only recurrence had significant worse median postrecurrence survival (7.4 and 8.3 months, respectively) compared with lung- or local-only recurrence (10.4 and 15.9 months, respectively).

**Conclusions:**

This study highlights that time to recurrence varies by location and significantly impacts postrecurrence survival outcomes. Multiple sites and liver-only recurrence had the poorest prognosis.

**Supplementary Information:**

The online version contains supplementary material available at 10.1245/s10434-026-19153-8.

Esophageal cancer is a major public health burden, responsible for more than 400,000 deaths annually worldwide.^1,2^ Treatment with curative intent for patients with locally advanced nonmetastatic esophageal adenocarcinoma typically involves neoadjuvant therapy followed by surgical resection.^3^ Despite advancements in treatment, a substantial proportion of patients, ranging from 36to 56%, experience disease recurrence posttreatment.^4,5^

Recurrent esophageal cancer can be difficult to manage due to its aggressive growth and multifocal recurrence patterns. Historically, many studies have grouped all patients with distant metastases together for recurrence and survival analysis in esophageal cancer.^6,7^ However, the biological behavior of recurrent disease varies; while many patients present with or progress to systemic disease, a subset develop isolated local recurrence or metastases confined to a single organ, such as the liver or lung. Stratification of recurrence patterns may reveal a novel perspective with respect to the biological behaviour and prognosis associated with recurrent esophageal adenocarcinoma.

Traditionally, management of recurrent esophageal cancer predominantly involves best supportive care or palliative chemotherapy.^7,8^ However, recent studies have begun to shed light on the potential benefits of more aggressive, tumor-directed therapies (i.e., surgical treatment or radiotherapy) for select patient groups compared with systemic chemotherapy alone.^9,10^ The prospect of improving long-term oncologic outcomes, and even cure, for highly selected patients with oligometastatic disease is gaining increasing traction internationally, reinforced by an increasing armamentarium of targeted systemic therapy options. This underscores the need for a more nuanced evaluation of recurrence patterns, time to recurrence, and prognosis to inform postoperative follow-up and enable more individualized treatment approaches.

As such, this study aims to provide a detailed insight in the pattern and timing of recurrence of esophageal adenocarcinoma after curative intent surgical resection. Additionally, this study aims to assess the distinct treatment and survival patterns associated with these recurrence locations within the framework of a large European collaborative multicenter cohort study.

## Methods

The present study represents a planned secondary analysis of the international multicenter ENSURE study, the details of which have been published previously.^11^ Consecutive patients undergoing surgery with curative intent for esophageal and esophagogastric junction cancer between 2009 and 2015 were included from 20 high-volume centers across Europe and Canada. Data were collected from prospectively maintained databases of the participating centers. Local approval of each corresponding institutional review board was obtained. The study was registered on ClinicalTrials.gov prior to inclusion of the first participant (NCT03461341). For the present study, only patients with adenocarcinoma of the esophagus or gastroesophageal junction were included. From the sampling cohort, patients with <30 days of postoperative follow-up were excluded to ensure sufficient time for recurrence to develop. The primary outcomes of interest were pattern of recurrence, time to recurrence, survival after recurrence, and treatment of patients with recurrence. Hence, patients with incomplete data regarding the initial pattern of recurrence were excluded.

### Data Collection, Follow-up and Recurrence

Patient and treatment-related parameters were collected from prospectively maintained databases of the participating centers and included demographics, comorbidities and performance status, histologic type, tumor location, clinical stage and grade, pathologic stage, treatment response and resection margins, neoadjuvant therapy details, operative details, overall postoperative morbidity, recurrence and associated treatment, and survival data.

Assessment of recurrence status varied among the included centers. All patients underwent postoperative follow-up, but the surveillance strategy differed: in 54.5% of patients, follow-up was routinely performed based primarily on clinical symptoms with imaging if indicated. In the remaining 45.5%, intensive surveillance was applied, including at least one annual CT or PET-CT scan. Most centers continued follow-up for at least 5 years postoperatively. Recurrence was confirmed by histopathological biopsy or clinical follow-up where possible, and for this specific study, only the initial number and sites of recurrences were evaluated.

Locoregional recurrence was defined as recurrence in the mediastinum, around the celiac trunk, former tumor bed or luminal recurrence at the anastomosis and did not include supraclavicular nodes. Distant recurrence was defined as all other recurrences not captured as locoregional and were categorized into five different groups: 1) liver only; 2) lung only; 3) other isolated distant metastases occurring in less common locations; 4) both isolated local recurrence and isolated distant recurrence to a single organ detected at the same time; 5) recurrences that occurred in multiple distant sites without locoregional involvement.

### Statistical Analysis

Categorical data are presented as numbers and percentages, and continuous data are expressed as mean ± standard deviation (SD) or median (range), as appropriate. Kaplan-Meier curves were used to assess recurrence-free survival and postrecurrence survival, and differences were evaluated by using the log-rank test. Recurrence-free survival (RFS) was defined as the time between the date of surgery and date of first recurrence, and postrecurrence survival (PRS) was defined as the time between the first recurrence and death or last follow-up. Statistical analysis was performed by using SPSS version 27.0 (IBM Corp., Armonk, NY). A *p*-value < 0.05 was considered statistically significant.

## Results

### Patient and Treatment-related Characteristics

From 3,299 patients who underwent surgery for esophageal adenocarcinoma and met the inclusion criteria, the median follow up was 60.4 months (range 2-122). Of these, 1,615 (48.9%) developed disease recurrence of whom 305 patients were excluded owing to insufficient data with regard to the specific recurrence patterns. In total, 1,310 patients with recurrent esophageal adenocarcinoma were included in this study. Patients without recurrence were not included, because the study specifically focused on recurrence patterns and postrecurrence survival. The distribution of patient and treatment-related characteristics are presented in Table 1.

**Table 1:** Patient, tumor, and treatment-related characteristics of patients with recurrent disease after esophagectomy with curative intent (n = 1,310)

Clinical characteristics		
	Value	%/SD
Gender		
Female	183	14%
Male	1,127	86%
Age (years)^a^	63.3	± 10.1
ASA grade		
1	350	26.7%
2	689	52.6%
3	269	20.5%
Neoadjuvant therapy		
None	214	16.3%
Chemotherapy	455	34.7%
Chemoradiotherapy	558	42.6%
Not specified	84	6.4%
Surgical approach		
Open	873	66.6%
Hybrid or total MIE	437	33.4%
Histologic grade		
Good	84	6.4%
Moderate	437	33.4%
Poor	557	44.0%
Unknown	212	16.2%
Pathologic T status^b^		
pT0	81	6.2%
pTis/T1	154	11.8%
pT2	181	13.8%
pT3	816	62.3%
pT4	75	5.7%
Pathologic N status^b^		
pN0	416	31.8%
pN1	307	23.4%
pN2	363	27.7%
pN3	224	17.1%
R0 resection^c^		
No	212	16.2%
Yes	1098	83.6%
Intensive surveillance^d^		
No	733	56%
Yes	577	44%

Data are numbers, with percentages in parentheses.^a^Expressed as mean ± SD; ^b^Classified according to the 8th edition of the AJCC staging manual; ^c^College of American Pathologists’ criteria. ^d^ Annual use of imaging during follow-up

Mean age was 63 years (SD ± 10.1), and most patients were male (n = 1127; 86%). Neoadjuvant therapy was administered in 958 (73%) patients. The surgical procedure consisted of an open approach in 873 (66.6%) and hybrid or total minimally invasive in 437 (33.4%) patients. The majority of patients had ≥pT3 tumors (n = 891, 68%) and positive lymph nodes (pN+, n = 894, 68%); 1,098 (83.6%) achieved a microscopically radical (R0) resection, as defined by the American College of Pathologists.^12^

### Recurrence Patterns

**Fig. 1:**
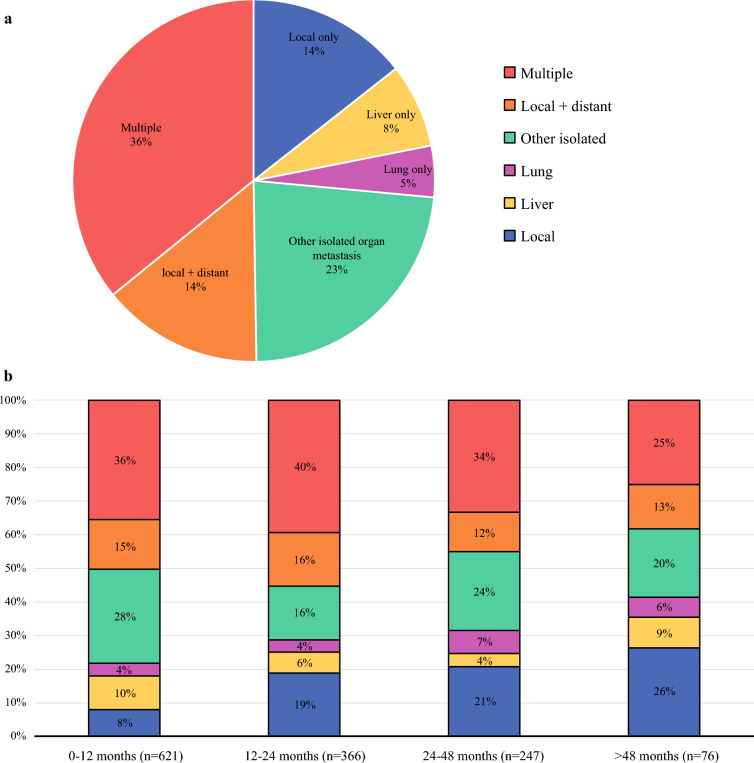
**a** Distribution of recurrence patterns. **b** Distribution of recurrence patterns at different time points

The recurrence patterns of the 1,310 included patients are presented in Fig. 1. Isolated locoregional recurrence occurred in 189 (14.4%) of the patients, of whom 62 had recurrence at the anastomosis. First recurrences isolated to a single distant site occurred in 463 (33.7%) patients, of whom liver (n = 98) and lung (n = 60) were the predominant organs. In the other 305 patients, recurrences occurred at other isolated sites, such as the peritoneum, adrenal, bone, brain, skin, and supraclavicular lymph nodes. A combination of local recurrence and isolated distant metastasis occurred in 189 (14.5%) patients, of whom liver (n = 45) and lung (n = 35) were also the predominant organs. In most patients (n = 469; 37.3%), recurrence was detected at multiple distant sites simultaneously.

Recurrence patterns were further examined in subgroup analyses based on tumor location, resection margin status, and neoadjuvant treatment modality. First, stratification by tumor location (esophageal vs. gastroesophageal junction adenocarcinoma, classified according to the AJCC 8th edition) showed no statistically significant difference in the distribution of recurrence types (*p* = 0.160). Second, although a slightly higher proportion of locoregional recurrence was observed in R1 resections compared with R0 (17.5% vs. 13.8%), this difference did not reach statistical significance (*p* = 0.079). Third, patients treated with surgery alone, neoadjuvant chemotherapy, or neoadjuvant chemoradiotherapy showed no statistically significant differences in recurrence distribution (*p* = 0.280). Detailed results of this analysis are provided in Supplementary Tables 1, 2 and 3, respectively.

### Time to Recurrence

Median RFS of the 1,310 included patients was 12.1 months (95% confidence interval [CI] 11.4–12.8) and 50, 76, and 89% of the recurrences occurred within 1, 2, and 3 years after surgery, respectively. After 5 years of follow-up, 97% of all recurrences had occurred. Among the patients with recurrence, 75% of the patients presented with symptoms and no difference in RFS between symptomatic and asymptomatic patients was found. In patients who underwent intensive postoperative surveillance, a difference was observed in the prevalence of symptoms among the different recurrence locations. Notably, liver and lung recurrences exhibited a lower percentage of symptomatic presentations at 22% and 30%, respectively, compared with the other types (range 46–68%).

The distribution of recurrence patterns at different time points is demonstrated in Fig. 1. At 1 year postesophagectomy, isolated locoregional recurrence was responsible for only 8% of all recurrences. By 2 and 4 years, this increased to 21 and 26% of the recurrences, respectively. However, distant metastasis remained the predominant pattern throughout, contributing 92% of recurrences in the first year after esophagectomy and decreasing to 74% by >4 years after esophagectomy.

**Fig. 2:**
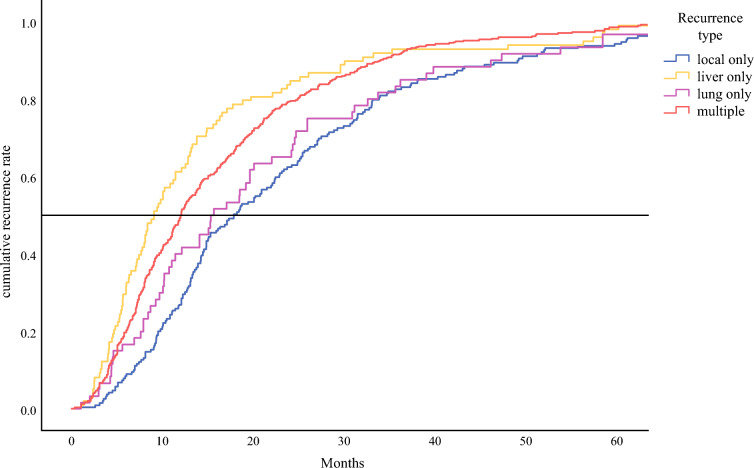
The cumulative recurrence rates, categorized by distinct recurrence patterns, are plotted over time. For clarity, the graph excludes the “other” recurrence category and merges the data for “local+distant” and “multiple” recurrences into a single curve. Median time to recurrence for the different recurrence locations, and *p*-values for difference are presented in Table 2

The median time to recurrence at each location is presented in Table 2. The cumulative recurrence rates over time per location are shown in Fig. 2. Local-only recurrence had the longest median RFS (17.8 months), followed by lung-only recurrence (15.2 months), combined local and single distant metastasis (11.9 months), multiple sites of recurrence (11.9 months), other isolated distant metastasis (9.6 months), and liver-only (9.0 months). Pairwise comparisons demonstrated that the median RFS of lung-only and local-only were significantly longer compared with all other recurrence locations (Table 2). There was no difference in median RFS between patients with local-only and lung-only recurrence.

**Table 2:** A pairwise comparison of median recurrence-free survival for the different recurrence locations

			*p*-values of pairwise comparison					
Recurrence type	n	Median RFS (months; 95% CI)	Local	Liver	Lung	Other isolated	Local & distant	Multiple
Locoregional	189	17.8 (14.2-21.2)	–	<0.01	0.46	<0.01	<0.01	<0.01
Liver	98	9.0 (7.3-10.7)	<0.01	–	0.01	0.56	0.24	0.18
Lung	60	15.2 (9.7-20.8)	0.46	0.01	–	0.04	0.04	0.03
Other isolated	305	9.6 (7.9-11-2)	<0.01	0.56	0.04	–	0.65	0.68
Local & distant	189	11.9 (11.0-13.4)	<0.01	0.24	0.04	0.65	–	0.96
Multiple	469	11.9 (11.0-12.9)	<0.01	0.18	0.03	0.68	0.96	–

*RFS* recurrence-free survival

### Postrecurrence Survival

**Fig. 3:**
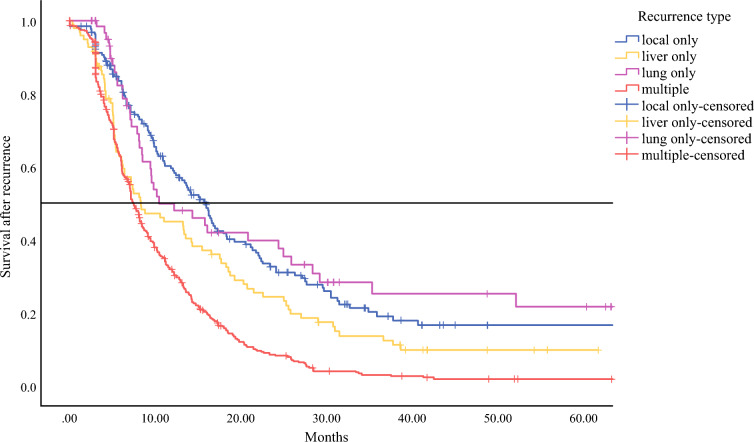
Survival rate after recurrence detection, categorized by the distinct recurrence pattern, plotted over time. For clarity, the graph excludes the “other” recurrence category and merges the data for “local+distant” and “multiple” recurrences into a single curve. Median postrecurrence survival for the different locations and *p*-values are presented in Table 3

Median overall survival from time of surgery for all patients with recurrence was 20.8 months (95% CI 19.4–22.3). Median PRS for all patients was 8.5 months (95% CI 7.9–9.0). Postrecurrence survival for the different location categories is presented in Table 3 and Fig. 3. Locoregional recurrence had the best median PRS (15.9 months), followed by lung-only recurrence (10.4 months). Postrecurrence survival for patients with recurrence to other or multiple organs ranged between 7 to 8 months. Patients with multiple recurrence sites had the shortest median PRS of 7 months. Pairwise comparisons for the specific recurrence locations demonstrated that the median PRS of local-only and lung-only was significantly longer compared to the other recurrence sites.

**Table 3:** Pairwise comparison of median postrecurrence survival for the different recurrence locations

			*p*-values of pairwise comparison					
Recurrence type	n	Median PRS (months; 95% CI)	Local	Liver	Lung	Other isolated	Local & distant	Multiple
Locoregional	189	15.9 (13.6-18.1)	–	<0.01	0.74	<0.01	<0.01	<0.01
Liver	98	8.3 (4.7-11.9)	<0.01	–	.026	.047	<0.01	<0.01
Lung	60	10.4 (3.2-17.6)	0.74	0.03	–	<0.01	<0.01	<0.01
Other isolated	305	7.8 (7.2-8.4)	<0.01	0.04	<0.01	–	0.72	0.15
Local & distant	189	8.4 (7.4-9.4)	<0.01	<0.01	<0.01	0.73	–	0.42
Multiple	469	7.4 (6.8-7.9)	<0.01	<0.01	<0.01	0.15	0.42	–

*PRS* postrecurrence survival

Additional analysis demonstrated that time to recurrence was a significant predictor of postrecurrence survival. The 1-year overall survival after recurrence detection for patients with a recurrence free survival of <12 months, 24–48 months, and >48 months was 29%, 44%, and 74%, respectively (*p* < 0.001).

### Treatment of Patients with Recurrence

The distribution of treatment types for each of the recurrence patterns are presented in Table 4. Palliative chemotherapy (with or without targeted therapy) was the most common treatment approach (33.6% of patients) for patients with recurrence. It demonstrated a median PRS of 12.3 months for all patients with recurrent disease, with notably high median PRS in liver-only recurrence (18.5 months). Best supportive care was the treatment approach for 31.5% of patients and exhibited lower median PRS, approximately 5.0 months for all patients with recurrence. Patients treated with radiation therapy only, representing 12.7% of recurrences, also experienced a relatively short PRS (median of 8.4 months). Chemoradiotherapy, used for 8.9% of patients with recurrence, demonstrated a reasonable PRS for local recurrence (median 27.6 months). Surgical resection, feasible in only 7.7% of patients, demonstrated the highest PRS across most recurrence types, peaking at median 65.4 and 58.0 months in local and lung recurrence, respectively. The category of other local cytoreductive therapies, accounting for a mere 1% of cases, lacked sufficient data for a conclusive PRS analysis.

**Table 4:** Distribution of various treatment types for the different recurrence patterns with corresponding survival after recurrence detection

Type of treatment	All recurrences (n = 1,310)		Local n = 189		Liver n = 98		Lung n = 60		Other isolated n = 305		Local & distant n = 189		Multiple n = 469	
	n (%)	PRS	n	PRS		PRS		PRS		PRS		PRS		PRS
Best supportive care	413 (31.5)	5.2 (4.9–5.4)	41 (22)	6.6 (5.1–8.1)	38 (39)	5.0 (3.8–6.2)	18 (30)	6.1 (4.6–7.6)	116 (38)	5.1 (4.5–5.8)	62 (33)	5.0 (4.7–5.3)	138 (29)	5.2 (4.8–5.5)
Palliative chemotherapy	440 (33.6)	12.3 (11.2–13.4)	56 (30)	16.5 (15.1–17.9)	32 (33)	18.5 (16.6–20.4)	24 (40)	10.4 (2.9–17.9)	65 (21)	11.1 (7.4–14.7)	79 (42)	11.4 (8.6–14.2)	184 (39)	11.1 (8.9–13.3)
Chemoradiotherapy	117 (8.9)	15.1 (13.0–17.1)	51 (27)	27.6 (17.1–38.1)	4 (4)	13.3 (6.6–26.4)	3 (5)	14.3 (0–28.8)	15 (5)	12.7 (6.2–19.3)	11 (6)	13.8 (8.5–19.0)	33 (7)	12.7 (11.5–14.0)
Radiation therapy only	167 (12.7)	8.4 (7.8–9.0)	26 (14)	16.1 (6.7– 25.6)	3 (3)	7.4 (7.3–7.4)	2 (3)	NA	45 (15)	8.1 (6.7–9.5)	24 (13)	8.4 (7.9–8.9)	67 (14)	7.5 (6.4–8.6)
Surgical resection of recurrence ± any other	101 (7.7)	27.5 (23.0–32.0)	6 (3)	65.4 (16.4–114)	5 (5)	37.7 (9.6–57.2)	9 (15)	58.0 (46.5–69.6)^a^	49 (16)	26.2 (13.3–38.9)	8 (4)	13.6 (7.7–28.8)	24 (5)	20.4 (5.9–34.8)
Other cytoreductive therapies	13 (1.0)	NA	0	NA	8 (8)	NA	0 (0)	NA	2 (1)	NA	1 (1)	NA	2 (1)	NA
Unknown	59 (4.5)		9 (5)		8 (8)		4 (7)		14 (4)		4 (2)		21 (5)	

*PRS* median postrecurrence survival in months with 95% confidence interval; *NA* not applicable. ^a^Reported as mean survival, because median was not reached

## Discussion

This international multicenter study offers comprehensive insights into the pattern, timing, survival, and treatment of initial recurrence after curative resection of esophageal adenocarcinoma. Results of the present study support the previously established poor prognosis of recurrent esophageal adenocarcinoma, with a median postrecurrence survival of 8.5 months. Although in agreement with some other reports, 8.5 months is relatively long compared with previous studies that reported median survival times after recurrence of approximately 3 months.^6,7,13–15^ This finding may be partly due to its inclusion of patients who received recent treatment at high-volume centres specializing in esophageal cancer care. Additionally, the implementation of intensive surveillance postsurgery in a significant proportion of these centres (46%) could lead to earlier detection of recurrences, potentially contributing to the observed prolongation of survival after recurrence due to lead time bias.^3^ Also consistent with previous findings, this study demonstrated that the majority of recurrences occur at distant sites (71%), associated with adverse survival compared with locoregional recurrence.^6,7,13–15^ It is likely that the observed difference in survival outcomes according to recurrence site reflects both differences in the underlying tumor biology, i.e., distant metastasis versus local recurrence, and differing treatment options.

While many previous studies have assessed outcomes among patients with distant recurrence as a single group, the large sample size and comprehensive recurrence data in this study enabled further stratification of six distinct recurrence patterns. Stratifying the different recurrence locations, rather than pooling them together, allows for a more nuanced understanding of recurrent esophageal adenocarcinoma. A key finding of this study is the variation in time to recurrence across different sites. Liver metastasis and isolated distant metastasis recurred most quickly after surgery (median of 9.0 and 9.6 months, respectively). In contrast, median recurrence free survival of lung-only (15.2 months) and local-only (17.8 months) recurrences were significantly longer compared to all other recurrence locations. At the same time, however, both liver and lung metastasis have limited symptom presentation at the time of recurrence detection.This disparity indicates that differences in time to recurrence detection may not be merely as a result of earlier symptom driven detection but may reflect the underlying differences in biological behaviour of the underlying disease or recurrence treatment offered in these patient groups.

This is also supported by the study's findings on PRS. While the median PRS was 8.5 months, there were significant differences based on the recurrence location. Patients with recurrence located in the liver or at multiple sites displayed a significantly shorter median PRS compared to those with isolated pulmonary or locoregional recurrence. In addition, this study demonstrated that a short time to recurrence was negatively associated with postrecurrence survival. These findings are consistent with data from ENSURE regarding the impact of intensive surveillance on survival outcomes.^3^ In the former analysis, overall survival was improved following intensive surveillance for those who underwent surgery only and those with lower pathological (y)pT stage. Together, these data suggest that patients with a more indolent and favourable disease biology, including those undergoing surgery alone who are chemotherapy naïve, and those with a major response to neoadjuvant therapy, may derive the greatest benefit from an intensified approach to surveillance and treatment of recurrent disease. Although the underlying biology of these phenomena are poorly understood, recognizing these differences in prognosis may enhance shared decision-making with respect to tailored surveillance strategies following curative intent surgery for esophageal adenocarcinoma.

The slower occurrence and better prognosis of local recurrence and solitary pulmonary recurrence has been recognized by previous studies.^16–18^ As such, these types of recurrence have been occasionally treated by targeted interventions such as surgery and (chemo)radiotherapy. In this context, some small non-randomized studies, and the present study demonstrate remarkably favourable long-term outcomes after resection—in a highly selected group of patients—of isolated pulmonary or local recurrence.^16,17^ Consistent with the present study, previous data suggest that the effect of tumor-directed therapy may be limited among patients with a shorter disease-free interval.^17^ Together, these findings suggest that patients with a longer disease-free interval may derive the greatest benefit from more radical approaches to the treatment of recurrent disease and that this parameter may help to inform clinical decision-making. Further research is needed to identify the selected group who benefit most from a radical approach to oligometastatic recurrence in this setting, as current therapeutic modalities are not without risk of added morbidity.

Liver-only and multiple-site recurrence on the other hand tended to occur quickly after curative intent surgery for esophageal adenocarcinoma and were associated with poor survival outcomes. Consistent with findings from earlier studies, this analysis demonstrated that patients who undergo palliative chemotherapy may achieve significantly greater postrecurrence survival time, with a median postrecurrence survival of 12.3 compared with approximately 5.0 months for those receiving best supportive care in this context.^7,19^ While the nonrandomized nature of these data limit the conclusions that can be drawn from this comparison, the present data highlight that where systemic therapy is feasible, median postrecurrence survival of approximately 1 year may be achieved. With the advent of immunotherapy, and the increasing armamentarium of targeted therapeutic options in recent years for patients with advanced disease, it is likely that outcomes for patients in the recurrent setting will continue to improve. These findings also highlight the importance of multidisciplinary care to facilitate recovery of performance status and overall wellbeing following curative intent treatment for esophageal cancer.^20^

This study contributes to the understanding of recurrent esophageal adenocarcinoma, complementing and expanding upon existing research in this domain. Understanding the impact of recurrence pattern and disease-free interval on postrecurrence prognosis may provide important data to inform the management of patients with recurrent disease. However, little is known about the biological mechanisms underlying differential recurrence patterns in this context. Certain molecular profiles may be associated with increased metastatic potential in esophageal adenocarcinoma.^21,22^ For example, the loss of SMAD4 has been linked with an increased risk of postoperative recurrence, as well as a shorter time to recurrence and reduced overall survival, whereas GATA4 amplification has also been associated with poor prognosis in esophageal cancer.^21,22^ Despite these advancements, the highly diverse mutational patterns in esophageal adenocarcinoma complicate the utilization of genetic information in current clinical practice for patient stratification and decision-making.^23^ Future research should also assess the impact of primary tumour and metastatic biomarker status on disease-free interval and postrecurrence survival to determine whether the presence of a targetable biomarker influences the benefit derived from intensified surveillance strategies or radical therapies in the context of recurrent disease. Recent data highlight the potential for emerging liquid biopsy techniques to identify the presence of micrometastatic disease among patients planned for treatment with curative intent.^24^ Future research may evaluate the use of ctDNA to guide the implementation of adjuvant therapies among patients at high risk of systemic recurrence, representing a new horizon in the management of esophageal adenocarcinoma. Integrating clinical and biomarker data within a molecular tumor board holds significant promise to advance personalized treatment approaches for patients with recurrent esophageal adenocarcinoma.

While this study provides valuable insights, a number of limitations should be acknowledged. Despite the comprehensive data collection from high-volume centers specializing in esophageal cancer, the variation in postoperative surveillance protocols across these centers may have affected both the timing and detection of recurrence. Nonetheless, this study captures outcomes associated with distinct patterns of recurrence, irrespective of whether this is identified through surveillance or symptomatic investigation. This study offers a cross-sectional view of recurrence after esophagectomy but does not provide a longitudinal assessment of the overall disease trajectory, because only the initial recurrence was registered and subsequent disease progression or treatment response following the first recurrence was not systematically tracked. In addition, the study lacked detailed information on the specific chemotherapy regimens administered, which precluded analysis of recurrence patterns by individual chemotherapy treatment protocols. Although detailed recurrence data were collected, the ENSURE database lacked detailed information about patient performance status at the time of recurrence, limiting the interpretation of factors influencing decision-making, particularly with respect to the utilization of best supportive care. Future research may incorporate longitudinal assessment of patient performance status, as well as symptoms associated with recurrent disease and associated changes in health-related quality of life.

## Conclusions

This large, international, multicenter study provides comprehensive insights into the pattern, timing, survival, and treatment of initial recurrence following curative resection of esophageal cancer. The results indicate that different recurrence patterns are associated with distinct times to recurrence. Moreover, the median survival after recurrence detection varies significantly between recurrence locations. In particular, patients with recurrence located at multiple sites or in the liver displayed a significant shorter median survival compared with those with isolated pulmonary or locoregional recurrence, whereas patients with a longer disease-free interval exhibited improved outcomes following treatment of recurrent disease. These findings imply differences in biological traits of the various recurrence patterns and could facilitate guidance in surveillance strategies, patient counseling, and treatment plans for recurrent disease.

## Electronic supplementary material

Below is the link to the electronic supplementary material.

Supplementary file1 (DOCX 16 KB)

## Appendix

See Table

**Table 5:** ENSURE study group

Country	Institution	Authors
Belgium	University Hospital Leuven, Leuven	Hans Van Veer, Lieven Depypere, Willy Coosemans, Philippe Nafteux
Canada	University of Toronto, Toronto	Paul Carroll, Frances Allison, Gail Darling
England	Churchill Hospital, Oxford University Hospitals NHS Foundation Trust	Serenydd Everden, Nicholas D Maynard
England	Derriford Hospital, Plymouth	Arun Ariyarathenam, Grant Sanders
England	East Sussex Healthcare NHS Trust	Shameen Jaunoo
England	Nottingham City Hospital	Pritam Singh, Simon Parsons, John Saunders, Ravinder Vohra
England	Queen Elizabeth Hospital, Birmingham	Aaditya Sinha, Benjamin HL Tan, John L Whiting
England	St. Mary's Hospital, London	Piers R Boshier, Sheraz R Markar, Giovanni Zaninotto, George B Hanna
England	The Newcastle Upon Tyne Hospitals NHS Foundation Trust	Alexander W Phillips, S Michael Griffin
England	Southampton General Hospital	Robert C Walker, Tim J Underwood
England	North Devon District Hospital, Barnstaple	John M Findlay
France	Centre Hospitalier Régional Universitaire de Lille	Guillaume Piessen
Germany	Klinikum rechts der Isar, TUM, Munich	Jorg Theisen, Hans Friess
Germany	University Hospital Cologne, Cologne	Christiane J Bruns, Wolfgang Schröder
Ireland	Galway University Hospital, Ireland	Chris G Collins, Oliver J McAnena, Siobhan Rooney, Aoife Quinn
Ireland	Mercy University Hospital, Cork	Conor Toale, Thomas J Murphy
Ireland	St. James's Hospital, Dublin	Jessie A Elliott, Narayanasamy Ravi, Claire L Donohoe, John V Reynolds
Italy	Azienda Ospedaliera di Padova	Marco Scarpa, Romeo Bardini, Silvia Degasperi, Luca Saadeh
Italy	Veneto Institute of Oncology IOV - IRCCS, Padua	Eleonora Pinto, Genny Mattara
Italy	Humanitas Research Hospital IRCCS Rozzano, Milan	Carlo Castoro, Rita Alfieri
Netherlands	Academic Medical Centre, Amsterdam	Marianne C Kalff, Suzanne S Gisbertz, Mark I Van Berge Henegouwen
Netherlands	Erasmus Medical Centre, Rotterdam	Sander JM van Hootegem, Sjoerd M Lagarde, J Jan B van Lanschot
Netherlands	University Medical Centre, Utrecht	B Feike Kingma, Lucas Goense, Jelle P Rurrda, Richard van Hillegersberg
Northern Ireland	Belfast Health and Social Care Trust, Belfast	Raymond Kennedy, P Declan Carey
Scotland	Royal Infirmary of Edinburgh, Edinburgh	Leanne Prodehl, Peter J Lamb, Richard JE Skipworth
Spain	Hospital Universitario Del Mar, Barcelona	Mariagiulia Dal Cero, Manuel Pera
Sweden	CLINTEC, Karolinska Institutet, Stockholm	Biying Huang, Fredrik Klevebro, Magnus Nilsson
Sweden	Karolinska Institutet, Department of Molecular Medicine and Surgery, Karolinska University Hospital, Stockholm	Asif Johar, Pernilla Lagergren
Sweden	Uppsala University Hospital, Uppsala	Gustav Linder, Magnus Sundbom
Switzerland	Lausanne University Hospital, Lausanne	Styliani Mantziari, Markus Schäfer, Nicolas Demartines

5.
