# Supplementary material for: Patterns, Timing, and Survival of Recurrence After Surgery for Esophageal and Junctional Adenocarcinoma in the European Multicentre ENSURE Study
Source: Ann Surg Oncol. 2026 Apr 6;33(7):6263–73. doi: 10.1245/s10434-026-19153-8 (PMC13242413; doi:10.1245/s10434-026-19153-8)
Supplement: Supplementary file 1 — Supplementary file1 (DOCX 16 KB) [file 10434_2026_19153_MOESM1_ESM.docx]

| Recurrence type | Junctional tumors | Esophageal tumors |
| --- | --- | --- |
| local only | 95 (13.9%) | 93 (14.9%) |
| liver only | 50 (7.3%) | 48 (7.7%) |
| lung only | 29 (4.3%) | 30 (4.8%) |
| other isolated (non)solid organ metastasis | 156 (22.9%) | 149 (23.9%) |
| local + distant | 116 (17.0%) | 72 (11.6%) |
| multiple | 236 (34.6%) | 231 (37.1%) |
| p-value for difference = 0.160 | | |

**Supplements**

**Supplemental Table 1. Distribution of Recurrence Patterns Stratified by Tumor Location (Esophageal vs. Gastroesophageal Junction Adenocarcinoma)**

**Supplemental Table 2. Distribution of Recurrence Patterns Stratified by Resection Margin Status** (R0 vs. R1)

| Recurrence type | R0 margin | R1 margin |
| --- | --- | --- |
| local only | 152 (13.8%) | 37 (17.5%) |
| liver only | 89 (8.1%) | 9 (4.2%) |
| lung only | 55 (5.0%) | 5 (2.4%) |
| other isolated (non)solid organ metastasis | 253 (23.0%) | 52 (24.5%) |
| local + distant | 152 (13.8%) | 37 (17.5%) |
| multiple | 397 (36.2%) | 72 (34.0%) |
| p-value for difference = 0.079 | | |

**Supplemental Table 3. Distribution of Recurrence Patterns Stratified by Neoadjuvant Treatment Modality**

| Recurrence type | Surgery alone (n = 214) | Neoadjuvant chemotherapy (n = 455) | Neoadjuvant chemoradiotherapy (n = 558) |
| --- | --- | --- | --- |
| local only | 29 (13.6%) | 65 (14.3%) | 80 (14.3%) |
| liver only | 21 (9.8%) | 29 (6.4%) | 38 (6.8%) |
| lung only | 10 (4.7%) | 19 (4.2%) | 28 (5.0%) |
| other isolated (non)solid organ metastasis | 48 (22.4%) | 110 (24.2%) | 131 (23.5%) |
| local + distant | 33 (15.4%) | 75 (16.5%) | 60 (10.8%) |
| multiple | 73 (34.1%) | 157 (34.5%) | 221 (39.6%) |
| p-value for difference = 0.280 | | | |
